# LncRNA ST7-AS1 is a Potential Novel Biomarker and Correlated With Immune Infiltrates for Breast Cancer

**DOI:** 10.3389/fmolb.2021.604261

**Published:** 2021-04-12

**Authors:** Ziwen Zhang, Han Zhang, Dongbo Li, Xiaoping Zhou, Jinlu Wang, Qingyuan Zhang

**Affiliations:** Department of Medical Oncology, Harbin Medical University Cancer Hospital, Harbin Medical University, Harbin, China

**Keywords:** lncRNA ST7-AS1, BRC, prognostic model, TCGA, biomarker, tumor microenvironment

## Abstract

**Background:** Long noncoding RNA (lncRNA) ST7-AS1 can be observed in various cancers, but its role in breast cancer (BRC) remains unclear. Our aim is to, on the basis of The Cancer Genome Atlas (TCGA) database, prove the correlation between lncRNA ST7-AS1 and BRC.

**Methods:** The lncRNA ST7-AS1 expression and its roles in the prognosis of BRC were explored using data from the TCGA database. The expression level of lncRNA ST7-AS1 in BRC samples was detected using RT-PCR. The 1-, 3-, or 5-year survival rate was predicted using a nomogram established through Cox proportional hazard regression. At last, the biological function was explored through gene ontology (GO) analysis and gene set enrichment analysis (GSEA). The hallmark pathways significantly involved in hub genes were described through functional enrichment analysis. The correlation between lncRNA ST7-AS1 expression and immune infiltration was analyzed through single-sample GSEA (ssGSEA).

**Results:** LncRNA ST7-AS1 expression was downregulated in BRC. Decreased lncRNA ST7-AS1 expression in BRC was correlated with advanced clinical pathologic characteristics (high grade, histological type, age, menopause status, and HER2 status), survival time, and poor prognosis. The nomogram was established for using lncRNA ST7-AS1 to predict 1-, 3-, or 5-year survival in patients with BRC. In addition, GO and pathway analyses suggested the involvement of lncRNA ST7-AS1 in cell cycle, DNA repair, and immune cell infiltration in the BRC immune microenvironment. We found the correlation of lncRNA ST7-AS1 with T helper cells and DC cells.

**Conclusion:** Low expression of lncRNA ST7-AS1 indicates poor prognosis and has an impact on cell cycle, DNA repair, and proportion of infiltrating immune cells in the BRC microenvironment. Therefore, lncRNA ST7-AS1 can be used as a protective prognostic marker and a potential treatment target for BRC.

## Introduction

Breast cancer is most commonly observed in female patients with cancer, which is a main cause of death induced by cancer. The proportion of BRC in all newly diagnosed cancers (Balasubramanian et al., 2019) in women reaches 30%, and cancers result in 15% of deaths in women (Siegel et al., 2019). The potential molecular mechanisms underlying tumor formation and progression which further complicate the effective treatment of BRC are poorly understood. Now, early diagnosis and prognosis can decrease the cancer-related mortality by some biomarkers, such as CEA and CA153, and image examinations (Mirabelli and Incoronato, 2013). As breast tumors are heterogeneous, BRC are classified according to their expression of key proteins ER, PR, HER2, etc., for individual therapy (Klinge, 2018). However, these biomarkers are not satisfactory for diagnosis or prognosis due to poor sensitivity and specificity. Thus, it is urgent to find effective prognostic biomarkers for diagnosing and treating BRC.

As a multiple class of transcripts, lncRNA has more than 200 bases and no protein-coding potential (Cech and Steitz, 2014). In tumors, some abnormal expressions of lncRNAs are important regulatory molecules that participate in the regulation of fundamental biological processes, affecting proliferation, apoptosis, metastasis, autophagy, etc. (Yang et al., 2014; Zeng et al., 2017; Chen et al., 2020). With more and more attention being paid to lncRNA as a tumor suppressor or oncogene in various tumors, lncRNA can be used as a therapeutic molecular target or a potential biomarker with prognostic values (Sun et al., 2017; Tian et al., 2017). LncRNA ST7-AS1 is a newly discovered one located on 7q31.2 (Qin et al., 2019). LncRNA ST7-AS1 with Copy Number Variations (CNVs) can cause gene disorder in downstream cancers, which is also strongly linked to proliferation, apoptosis, and cell migration belonging to the biological processes of cancer (Xu et al., 2019). Based on current evidence, ST7-AS1 has a strong correlation with human glioma and laryngeal squamous cell carcinoma (Liu et al., 2015; Tian et al., 2017). However, the functions of lncRNA ST7-AS1 in tumors remain unknown.

Here, we presented lncRNA ST7-AS1 as a positive prognostic biomarker for BRC patients in public databases such as TCGA. We also investigated the distinctive genomic alterations and functional networks associated with lncRNA ST7-AS1 expression and evaluated its role in tumor immunity. Our work could potentially reveal more direct evidence for hypothesizing lncRNA ST7-AS1 expression as a potential treatment target or a prognostic biomarker in risk stratification and provide insights into the molecular mechanisms for BRC patients.

## Methods

### RNA Sequencing Data and Clinical Data From the TCGA Database

We obtained the lncRNA ST7-AS1 expression (1,065 cases for the workflow type: HTSeq-FPKM and HTSeq-Counts) and clinical data of BRC projects from TCGA. Those without RNA sequencing data and an overall survival of at least 30 days were excluded. We then converted level 3 HTSeq-FPKM data into TPM (Transcripts Per Million) reads for subsequent analyses. As shown in Table 1, the data of 1,065 patients were summarized. We compared the expression data (HTSeq-Counts) of the high lncRNA expression group and the low lncRNA expression group and identified differentially expressed genes (DEGs) using the DESeq2 R package (Love et al., 2014), with thresholds of |log 2-fold change (FC)| > 1.5 and adjusted *p* value < 0.05.

**TABLE 1:** Association between lncRNA ST7-AS1 expression and clinicopathologic features in the validation cohort.

Characters	Level	Low expression of ST7-AS1	High expression of ST7-AS1	*p*	Test
N		533	532		
T stage (%)	T1	118 (22.2%)	157 (29.6%)	0.002	
	T2	325 (61.1%)	290 (54.7%)		
	T3	64 (12.0%)	73 (13.8%)		
	T4	25 (4.7%)	10 (1.9%)		
N stage (%)	N0	245 (46.9%)	262 (50.0%)	0.419	
	N1	175 (33.5%)	174 (33.2%)		
	N2	66 (12.6%)	50 (9.5%)		
	N3	36 (6.9%)	38 (7.3%)		
M stage (%)	M0	470 (97.3%)	419 (98.4%)	0.396	
	M1	13 (2.7%)	7 (1.6%)		
Pathologic stage (%)	Stage I	82 (15.6%)	98 (18.9%)	0.116	
	Stage II	299 (57.1%)	307 (59.3%)		
	Stage III	131 (25.0%)	107 (20.7%)		
	Stage IV	12 (2.3%)	6 (1.2%)		
PR status (%)	Negative	169 (34.0%)	169 (32.8%)	0.738	
	Positive	328 (66.0%)	346 (67.2%)		
ER status (%)	Negative	108 (21.7%)	129 (25.0%)	0.248	
	Positive	390 (78.3%)	388 (75.0%)		
HER2 status (%)	Negative	269 (73.3%)	279 (82.5%)	0.004	
	Positive	98 (26.7%)	59 (17.5%)		
PAM50 (%)	Basal	82 (15.4%)	108 (20.3%)	<0.001	
	Her2	52 (9.8%)	30 (5.6%)		
	LumA	249 (46.7%)	302 (56.8%)		
	LumB	141 (26.5%)	61 (11.5%)		
	Normal	9 (1.7%)	31 (5.8%)		
Histological type (%)	Infiltrating ductal carcinoma	418 (85.8%)	339 (71.8%)	<0.001	
	Infiltrating lobular carcinoma	69 (14.2%)	133 (28.2%)		
Race (%)	Asian	33 (7.0%)	27 (5.4%)	0.059	
	Black or African American	73 (15.5%)	106 (21.0%)		
	White	366 (77.5%)	371 (73.6%)		
Menopause status (%)	Peri	18 (3.8%)	21 (4.4%)	0.734	
	Post	353 (73.5%)	340 (71.4%)		
	Pre	109 (22.7%)	115 (24.2%)		
Anatomic neoplasm subdivisions (%)	Left	287 (53.8%)	266 (50.0%)	0.232	
	Right	246 (46.2%)	266 (50.0%)		
TP53 status (%)	Mut	179 (36.4%)	156 (33.6%)	0.408	
	WT	313 (63.6%)	308 (66.4%)		
PIK3CA status (%)	Mut	172 (35.0%)	142 (30.6%)	0.172	
	WT	320 (65.0%)	322 (69.4%)		
Age (median [IQR])		59.00 [49.00,68.00]	58.00 [48.00,66.00]	0.179	Nonnorm

Next, we used a total of 15 PCR samples of BRC patients admitted to the Harbin Medical University Cancer Hospital from February 2020 to June 2020. The study protocol has obtained the approval of the Ethics Committee of Harbin Medical University Cancer Hospital and is in conformity with the Declaration of Helsinki.

### RNA Isolation and Quantitative RT-PCR

Based on the manufacturer’s protocol, we used the Trizol reagent (Invitrogen) to isolate total RNA from clinical samples. Total RNA was reversely transcribed into cDNA for PCR amplification. By using the SYBR Green I real-time detection kit (Cwbio, Beijing, China), we performed real-time quantitative PCR (RT-PCR) on a CFX96 Detection System (Bio-Rad, California, United States). Specific primers for lncRNA ST7-AS1 and GAPDH were as follows: LncRNA ST7-AS1 forward primers: 5′-ACC CTA CTC TGC CTC CCT TAT C-3′, reverse primers: 5′-TAG CAT CTG CCA CCC AAA TC-3′; GAPDH forward primers: GAA GGT GAA GGT CGG AGT CA, reverse primers: TTG AGG TCA ATG AAGG GGTC.

### Nomogram Construction and Evaluation

Based on the multivariate Cox analysis results, we established a nomogram to predict the prognosis of BRC patients. According to the prognosis model, we calculated each patient’s risk score as the total score of each parameter, which could predict the prognosis of BRC patients. The accuracy estimation of nomogram prediction was obtained from a calibration plot. It was found that the bias-corrected line in the calibration plot was close to the ideal curve (Keynesian cross), indicating a strong consistency between predicted values and observed values. The nomogram discrimination was determined using a concordance index (C-index), and 1,000 resamples were used in calculation by bootstrap approach. In this study, all statistical tests were two-tailed, with a statistical significance level of 0.05.

### Enrichment Analysis

The GO and Kyoto Encyclopedia of Genes and Genomes (KEGG) pathway enrichment analyses were performed for high and low lncRNA ST7-AS1 expression groups on the DEGS using the clusterProfiler package (Yu et al., 2012).

### Gene Set Enrichment Analysis

Using the clusterProfiler package (Yu et al., 2012), the subtype specific gene expression patterns and potential cellular pathways were elucidated on GSEA software (Subramanian et al., 2005). Based on the lncRNA ST7-AS1 expression level, we divided gene expression data into high lncRNA ST7-AS1 and low lncRNA ST7-AS1 groups, and each analysis included 1,000 times of gene set permutations. A function or pathway term with a false discovery rate (FDR) of less than 0.25 and a *p* value of less than 0.05 was considered statistically significant.

### Immune Infiltration Analysis by ssGSEA

We used a GSVA R package to quantify the BRC immune infiltration of 24 tumor-infiltrating immune cells in tumor samples through ssGSEA. According to the 509 gene signatures of 24 tumor-infiltrating lymphocytes (TILs) (Bindea et al., 2013), comprising natural killer (NK) cell, T follicular helper cells (Tfhs), CD56 bright NK, CD56dim NK, central memory CD4^+^ T cell, macrophages, cytotoxic cells, dendritic cells (DCs), CD8^+^ B cells, effector memory T cell (Tem), eosinophils, gamma delta T cells, activated DC, immature DC, mast cells, neutrophils, plasmacytoid DC, T helper cell, regulatory T cells (Tregs), type-1 T helper cells (Thp1), Th2, and Th17, the relative enrichment score of every immunocyte was quantified. The correlation between lncRNA ST7-AS1 and infiltration levels of immune cells was analyzed by the Spearman correlation, and these immune cells with the different expression groups of lncRNA ST7-AS1 were analyzed by the Wilcoxon rank sum test.

### Analysis of Protein–Protein Interactions Network

Search Tool for the Retrieval of Interacting Genes (STRING; https://string-db.org/) is an online database for predicting functional interactions between proteins. The cut-off criterion we used was an interaction score > 0.4. Hub genes were extracted from the PPI network using the Molecular Complex Detection (MCODE) algorithm (Bader and Hogue, 2003) so as to identify crucial subnetworks.

### Statistical Analysis

R (Version 3.5.1) was used for all statistical analyses and plots. The correlations between clinicopathological characteristics and lncRNA ST7-AS1 expression were evaluated using the Chi-squared test, Fisher exact test, Kruskal–Wallis (KW) test, Wilcoxon signed-rank test, Wilcoxon rank sum test, and logistic regression. Kaplan-Meier method was adopted to draw survival curves [hazard ratio (HR), 95% CI]. Through univariate and multivariate analysis combined with Cox logistic regression models, other clinical factors impacting the survival and the lncRNA ST7-AS1 expression level were found. All reported *p* values were two-sided, and those less than 0.05 had statistical significance in all tests. The discrimination ability of lncRNA ST7-AS1 in BRC was evaluated through receiver operating characteristic (ROC) analysis using the pROC package (Robin et al., 2011).

## Results

### LncRNA ST7-AS1 Expression in BRC Patients From TCGA

**FIGURE 1:**
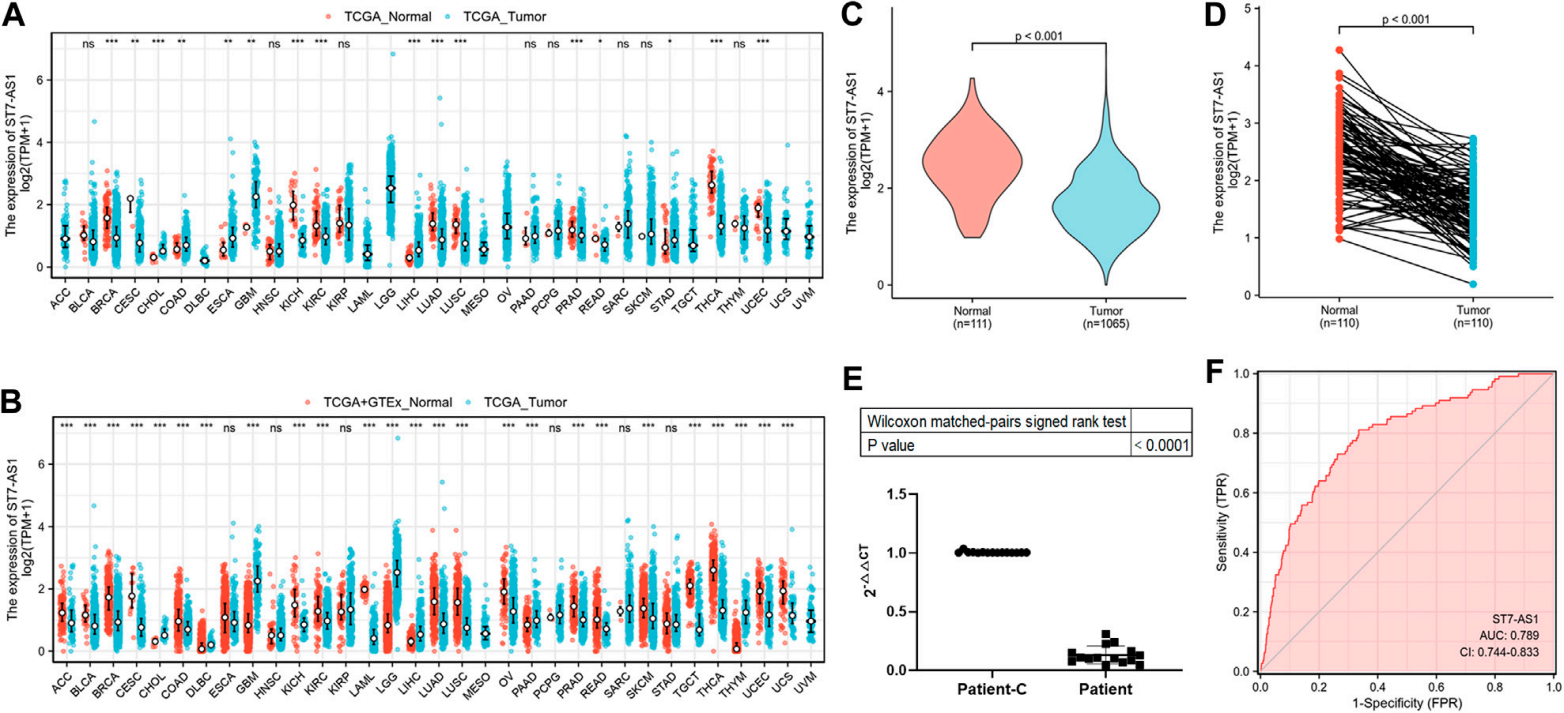
LncRNA ST7-AS1 expression in patients with BRC. Expression of lncRNA ST7-AS1 in different types of tumors in the TCGA database **(A)** and in TCGA + GTEx **(B)**. Expression levels of lncRNA ST7-AS1 were shown in nonpaired tumor tissues and adjacent normal tissues **(C)** and paired samples **(D)**. **(E)** Analysis of 15 patients’ samples by RT-PCR showed that, compared with paired normal breast tissues (Patient-C), cancer tissues (Patient) had a lower expression of lncRNA ST7-AS1. **(F)** ROC curves with lncRNA ST7-AS1 expression in TCGA.

To have a better understanding of the correlation of lncRNA ST7-AS1 expression in cancer, we showed the lncRNA ST7-AS1 across all TCGA differential expressions between tumor and adjacent normal tissues (Figure 1A) or normal tissue (Figure 1B). To further evaluate the difference of lncRNA ST7-AS1 expression in BRC, 1,065 patients’ clinical and gene expression data were downloaded from TCGA (Table 1). We used the Wilcoxon rank sum test to compare the lncRNA ST7-AS1 expression between BRC samples and adjacent normal tissues. Compared with 1,065 BRC tissues, lncRNA ST7-AS1 had a high expression in 111 healthy tissues (*p* < 0.001) (Figure 1C), and lncRNA ST7-AS1 expression in 111 paired normal breast tissues was greatly higher compared with that in 111 tumor tissues (*p* < 0.001) (Figure 1D). Based on the observation results of 15 patients’ samples by RT-PCR, BRC tissues had a lower lncRNA ST7-AS1 expression level compared with paired normal breast tissues (Figure 1E). ROC analysis was conducted on data of tumor tissues and normal ones to measure the discrimination value of lncRNA ST7-AS1. The AUC = 0.789 (Figure 1F).

### LncRNA ST7-AS1 Expression Correlated With Clinicopathologic Features

**FIGURE 2:**
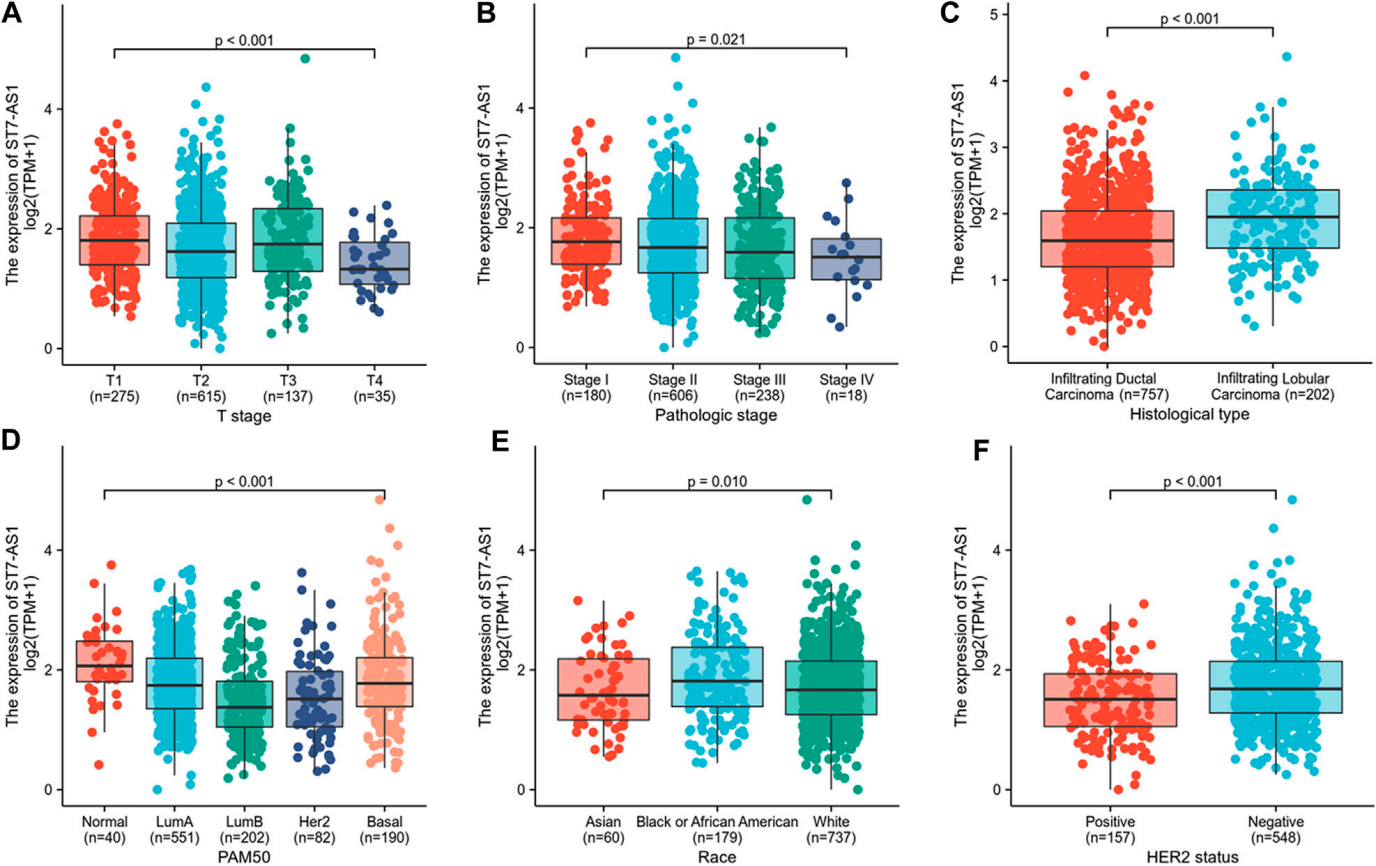
Correlation with lncRNA ST7-AS1 expression and clinicopathologic characteristics. Correlation with lncRNA ST7-AS1 expression and clinicopathologic characteristics, including **(A)** T stage, **(B)** pathological stage, **(C)** histological type, **(D)** PAM50 **(E)** race, and **(F)** HER2 status.

In the cohort, 532 cases showed high lncRNA ST7-AS1 expression and 533 cases showed low ST7-AS1 expression. Correlation analysis was carried out to identify the clinicopathologic features and ST7-AS1 expression level. As shown in Table 1 and Figure 2, reduced expression of ST7-AS1 was significantly correlated with the grade of topography distribution (*p* < 0.001), pathological stage (*p* = 0.0021), histological type (*p* < 0.001), race (*p* = 0.001), HER2 status (*p* < 0.001), and PAM50 (*p* < 0.001).

According to univariate analysis using logistic regression, as a categorical dependent variable, the expression of lncRNA ST7-AS1 (based on the median value) was correlated with poor clinicopathological prognosis. Decreased lncRNA ST7-AS1 expression in BRC was significantly correlated with pathologic stage (OR = 0.74 for Stage III/IV vs. Stage I/II, *p* = 0.04), HER2 status (OR = 0.58 for positive vs. negative, *p* = 0.003), and histological type (OR = 2.38 for infiltrating lobular carcinoma vs. infiltrating ductal carcinoma, *p* < 0.001) (Table 2). According to the results, BCR with low expression of lncRNA ST7-AS1 is prone to developing into poor outcomes.

**TABLE 2:** LncRNA ST7-AS1 expression associated with clinical pathological characteristics (logistic regression).

Characteristics	Total (N)	Odds Ratio (OR)	*p* value
T stage (T3 & T4 vs. T1 & T2)	1,062	0.92 (0.67–1.28)	0.636
N stage (N1 & N2 & N3 vs. N0)	1,046	0.88 (0.69–1.13)	0.321
M stage (M1 vs. M0)	909	0.60 (0.23–1.49)	0.287
Pathologic stage (Stage III & Stage IV vs. Stage I & Stage II)	1,042	0.74 (0.56–0.99)	0.040
PR status (positive vs. negative)	1,012	1.05 (0.81–1.37)	0.689
ER status (positive vs. negative)	1,015	0.83 (0.62–1.11)	0.219
HER2 status (positive vs. negative)	705	0.58 (0.40–0.83)	0.003
Histological type (infiltrating lobular carcinoma vs. infiltrating ductal carcinoma)	959	2.38 (1.72–3.30)	<0.001
TP53 status (Mut vs. WT)	956	0.89 (0.68–1.16)	0.371
PIK3CA status (Mut vs. WT)	956	0.82 (0.63–1.08)	0.152

We used Cox model to conduct the univariate analysis of prognostic factors for OS. There was a correlation between low expression of lncRNA ST7-AS1 and poor OS (*p* = 0.001, HR = 0.576 [95% CI 0.414–0.801]). In addition, there was a correlation between higher TNM stage (T: *p* = 0.007, HR = 1.673 [95% CI 1.152–2.429]; N: *p* < 0.001, HR = 2.145 [95% CI 1.497–3.073]; M: *p* < 0.001, HR = 4.327, 95% CI [2.508–7.465]), higher pathologic stage (*p* < 0.001, HR = 2.519 [95% CI 1.787–3.549]), older patients (*p* < 0.001, HR = 2.036 [95% CI 1.468–2.822]), and postmenopause status (*p* < 0.001, HR = 2.405 [95% CI 1.445–4.002]) and poor OS (Table 3). Then, we used Cox regression model for multivariate analysis. In multivariate analysis, the lncRNA ST7-AS1 expression (*p* = 0.037, HR = 0.541 [95% CI 0.304–0.962]), pathologic stage (*p* = 0.036, HR = 2.411 [95% CI 1.058–5.493]), ER status (*p* < 0.001, HR = 2.931 [95% CI 1.632–5.262]), and age (*p* < 0.001, HR = 3.388 [95% CI 1.743–6.584]) had an independent correlation with OS (Table 3).

**TABLE 3:** Univariate regression and multivariate survival model of prognostic covariates in patients with BRC.

	Total(N)	HR (95% CI) univariate analysis	*p* value univariate analysis	HR (95% CI) multivariate analysis	*p* value multivariate analysis
T stage (T3 & T4 vs. T1 & T2)	1,061	1.673 (1.152–2.429)	0.007	2.102 (0.976–4.528)	0.058
N stage (N1 & N2 & N3 vs. N0)	1,045	2.145 (1.497–3.073)	<0.001	1.315 (0.653–2.648)	0.443
M stage (M1 vs. M0)	909	4.327 (2.508–7.465)	<0.001	2.121 (0.636–7.075)	0.221
Pathologic stage (Stage III & Stage IV vs. Stage I & Stage II)	1,041	2.519 (1.787–3.549)	<0.001	2.411 (1.058–5.493)	0.036
PR status (negative vs. positive)	1,011	1.312 (0.931–1.849)	0.120		
ER status (negative vs. positive)	1,014	1.420 (0.983–2.052)	0.062	2.931 (1.632–5.262)	<0.001
HER2 status (negative vs. positive)	705	0.621 (0.378–1.019)	0.059	1.303 (0.661–2.566)	0.445
Age (>60 vs. <=60)	1,064	2.036 (1.468–2.822)	<0.001	3.388 (1.743–6.584)	<0.001
Race (White vs. Asian & Black or African American)	975	0.880 (0.593–1.306)	0.526		
Histological type (infiltrating lobular carcinoma vs. infiltrating ductal carcinoma)	959	0.860 (0.546–1.355)	0.516		
Anatomic neoplasm subdivisions (right vs. left)	1,064	0.776 (0.559–1.077)	0.130		
Menopause status (post vs. pre & peri)	955	2.405 (1.445–4.002)	<0.001	2.374 (0.986–5.714)	0.054
TP53 status (Mut vs. WT)	955	1.218 (0.858–1.730)	0.269		
PIK3CA status (Mut vs. WT)	955	1.015 (0.696–1.479)	0.938		
ST7-AS1 (high vs. low)	1,064	0.576 (0.414–0.801)	0.001	0.541 (0.304–0.962)	0.037

### LncRNA ST7-AS1 Expression Is Survival-Associated

**FIGURE 3:**
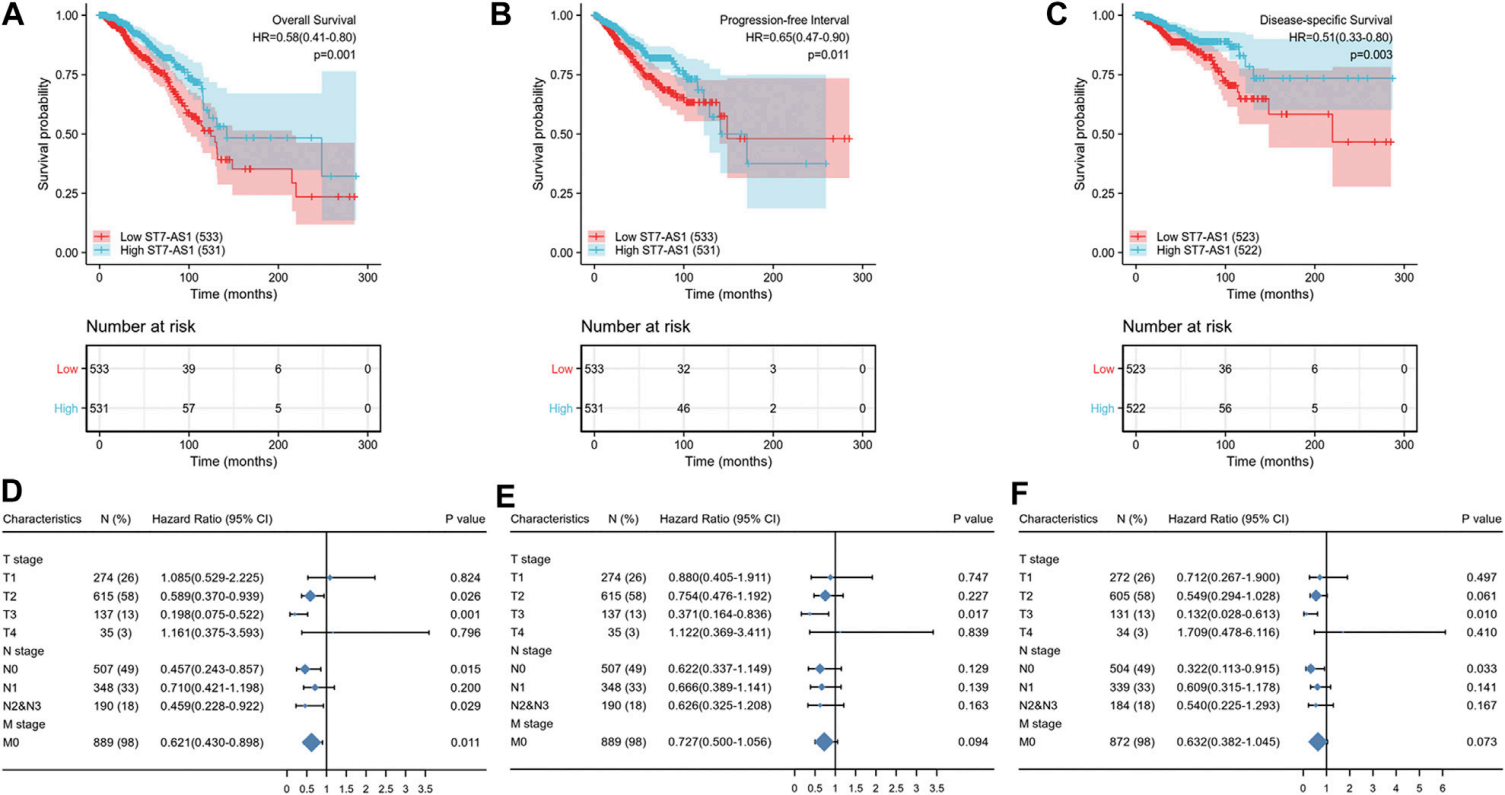
Association between lncRNA ST7-AS1 expression and BRC patients’ outcomes. **(A)** for OS, **(B)** for PFS, and **(C)** for DSS. The forest plot showed the impacts of expression of lncRNA ST7-AS1 in the presence of TNM stages for **(D)** OS, **(E)** PFS, and **(F)** DSS.

Based on the median expression of lncRNA ST7-AS1, the cohorts included the low expression subgroup and the high expression subgroup. Based on the KM Plotter in TCGA, the OS of patients with high expression level of ST7-AS1 was significantly longer than that of patients with low expression level of ST7-AS1 [HR = 0.58 (0.41–0.80), *p* = 0.001] (Figure 3A). Compared with patients with high expression level of ST7-AS1, BRC patients with low expression level of ST7-AS1 had an obvious lower progression-free survival (PFS) (HR = 0.65, CI 0.47–0.90, *p* = 0.011) (Figure 3B). Similarly, compared with the low expression group, the high expression group had an obvious higher disease-specific survival (DSS) (HR = 0.51, CI 0.33–0.80, *p* = 0.003) (Figure 3C). Moreover, in the presence of TNM stages, multivariable hazards models were used to evaluate the impacts of expression of lncRNA ST7-AS1 on OS, PFS, and DSS, respectively (Figures 3D–F).

**FIGURE 4:**
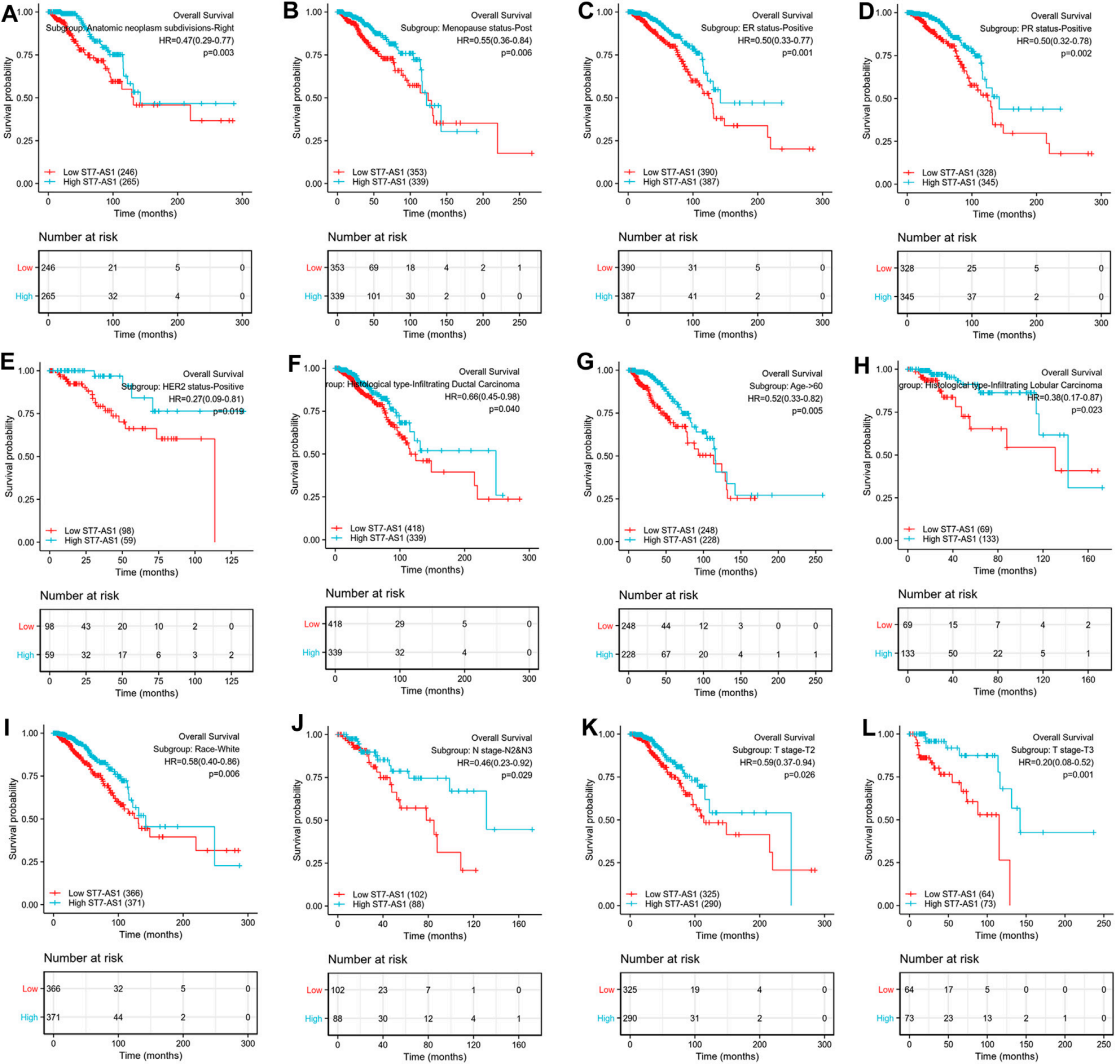
Prognostic value of differential expression of lncRNA ST7-AS1 in different subgroups. Prognostic value of differential expression of lncRNA ST7-AS1 in different subgroups, including **(A)** anatomic neoplasm subdivision-right, **(B)** menopause status-post, **(C)** ER-positive, **(D)** PR-positive, **(E)** HER2-positive, **(F)** infiltrating ductal carcinoma, **(G)** infiltrating lobular carcinoma, **(H)** age>60° years, **(I)** race-white, **(J)** N stage N2 and N3, **(K)** T stage-T2, and **(L)** T stage-T3.

Prognostic values of differential expression of lncRNA ST7-AS1 in different subgroups, including anatomic neoplasm subdivision-right, menopause status-post, ER-positive, PR-positive, HER2-positive, infiltrating ductal carcinoma, infiltrating lobular carcinoma, age >60°years, race-white, M stage-M0, N stage N2 and N3, T stage-T2, T stage-T3 (all *p* < 0.05), are shown in Figure 4. The above data indicated decreased lncRNA ST7-AS1 level is associated with poor prognosis.

### Construction and Validation of LncRNA ST7-AS1 Based Nomogram

**FIGURE 5:**
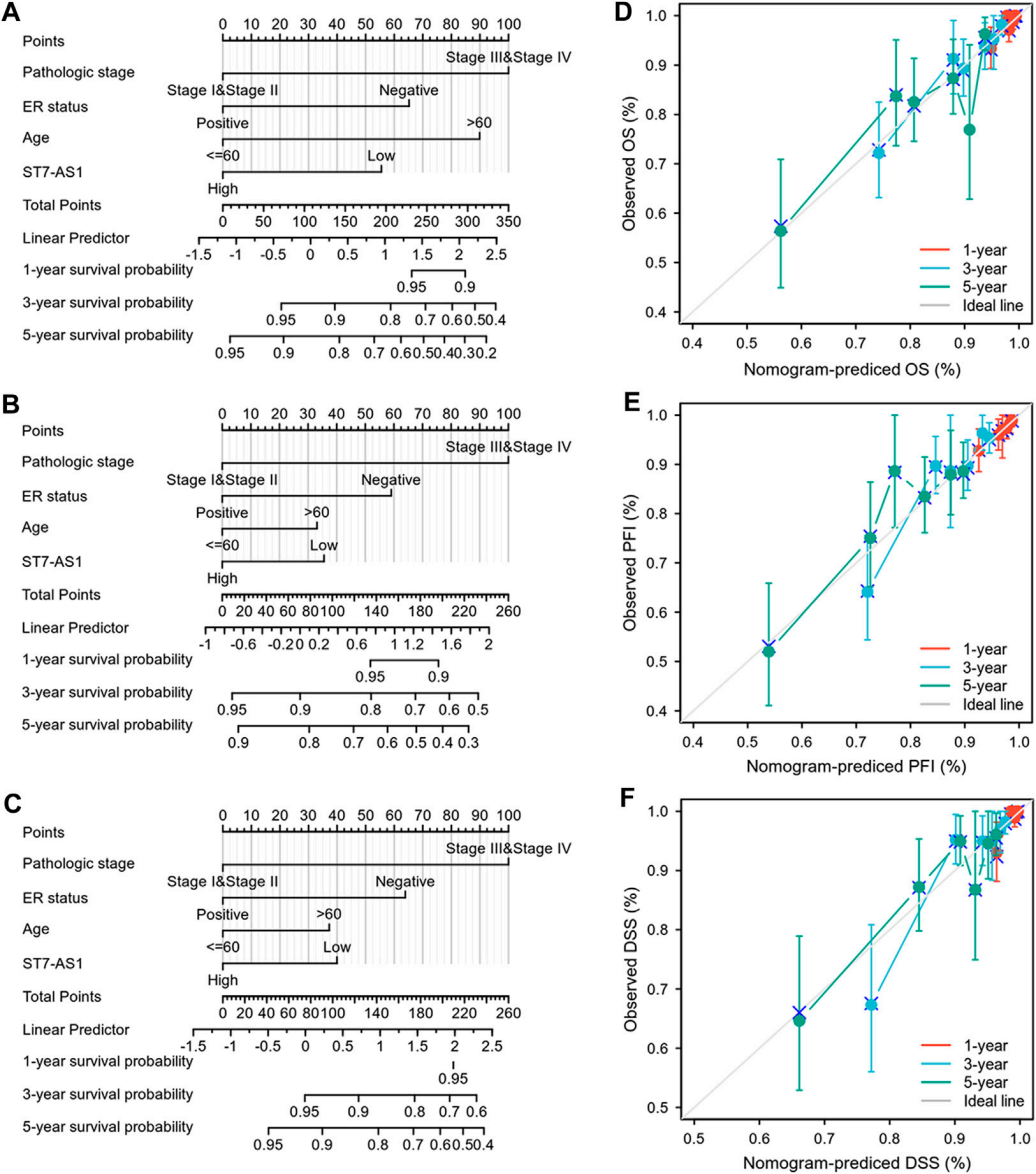
Construction and performance validation of the lncRNA ST7-AS1-based nomogram for BRC patients. Nomogram to predict **(A)** OS, **(B)** PFI, and **(C)** DSS for BRC patients. The calibration curve and Hosmer–Lemeshow test of nomograms in the TCGA-BRC cohort for **(D)** OS, **(E)** PFI, and **(F)** DSS.

In order to provide clinicians with predicted prognosis of BRC patients quantitatively, we established the nomogram combining lncRNA ST7-AS1 and independent clinical risk factors (pathologic stage, ER status, and age). Higher total points on the nomogram for OS, progression-free interval (PFI), and DSS, respectively, indicated a worse prognosis (Figures 5A–C).

Based on the calibration curve of nomograms for OS, PFI, and DSS, the predictions conformed well to observations in all patients, and the test showed no deviation from the perfect fit. The nomogram had a C-index of 0.750 and contained 1,000 bootstrap replicates (95% CI: 0.727–0.773) for OS. We also found PFS (C-index: 0.703, CI: 0.676–0.730) and DSS (C-index: 0.778, CI: 0.747-0.809). It was found that the bias-corrected line in the calibration plot was close to the ideal curve (Keynesian cross), indicating a strong correlation between predicted values and observed values (Figures 5D–F). In summary, these results indicate that the nomogram can well predict short- or long-term survival of BRC patients.

### Potential Mechanism of LncRNA ST7-AS1 in Regulating the Progression of BRC

**FIGURE 6:**
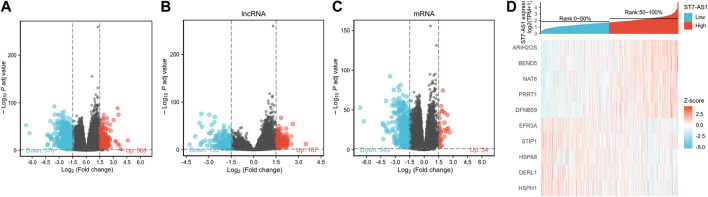
Identifying differentially expressed genes between high and low expression of lncRNA ST7-AS1 groups. **(A)** Volcano plot of differential gene profiles between lncRNA ST7-AS1 high expression and lncRNA ST7-AS1 low expression. **(B)** lncRNA and **(C)** mRNA profiles between lncRNA ST7-AS1 high expression and lncRNA ST7-AS1 low expression. **(D)** Hot map of the top 10 DEGs between lncRNA ST7-AS1 high expression and lncRNA ST7-AS1 low expression.

The DESeq2 package was used to analyze the data from TCGA in R (adjusted *p* value < 0.05, |log2 FC|>1.5). We identified 1,104 DEGs (including 568 and 578 upregulated and downregulated, respectively), 369 differentially expressed lncRNAs (including 187 and 182 upregulated and downregulated, respectively), and 377 differentially expressed mRNAs (including 34 and 343 upregulated and downregulated, respectively) in total (Figures 6A–C). Relative expression values for the top 10 DEGs between high and low lncRNA ST7-AS1 expression groups are illustrated in Figure 6D.

**FIGURE 7:**
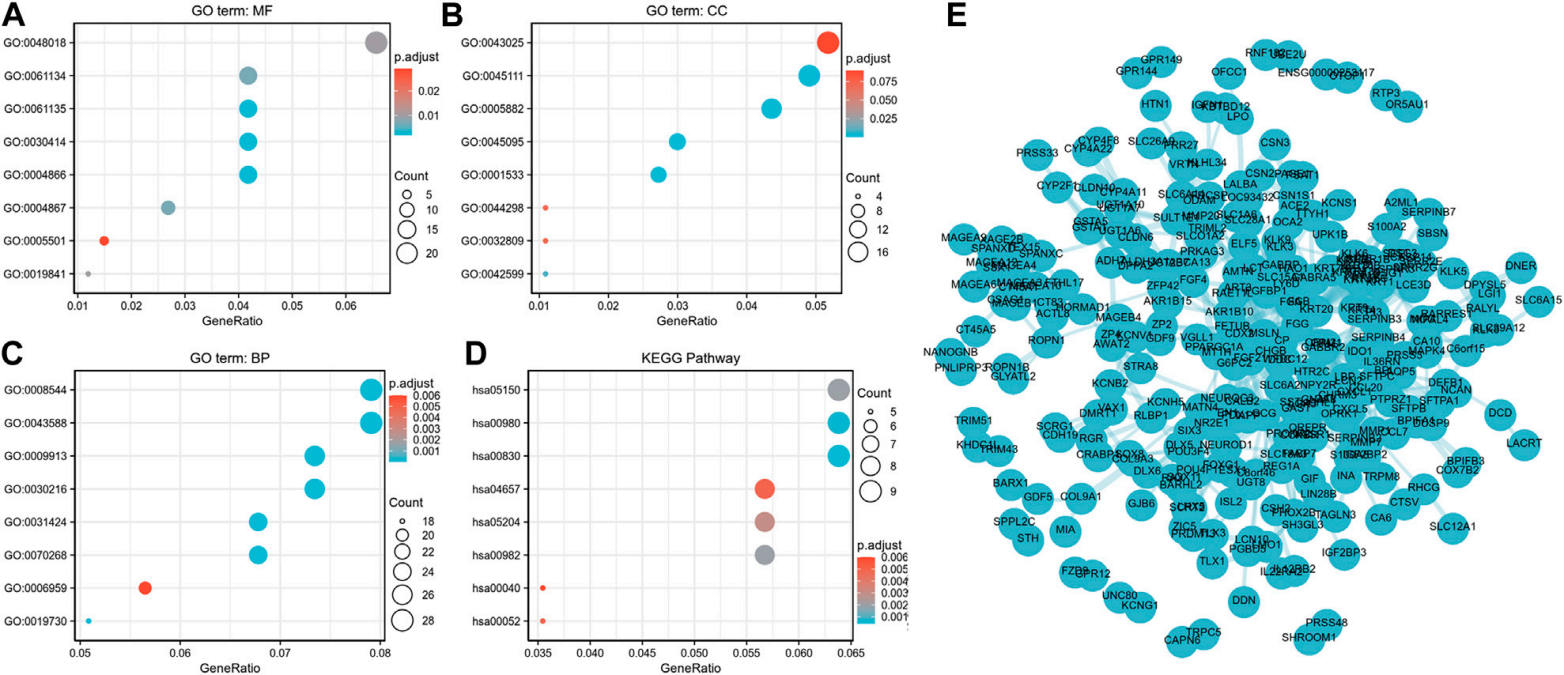
Functional annotation and prediction of signaling pathways. **(A–C)** GO enrichment analyses and **(D)** KEGG analysis of genes were conducted and displayed in bubble chart. **(E)** The protein–protein interaction network of DEGs.

ClusterProfiler was used for GO and KEGG enrichment analyses of the functions of lncRNA ST7-AS1 associated DEGs in BRC. The top GO enrichment items were classified into three functional groups. Molecular functions (MF) mainly involve receptor ligand activity, peptidase regulator activity, endopeptidase regulator activity, peptidase inhibitor activity, and endopeptidase inhibitor activity. The cellular components (CC) were mainly transcription regulation by intermediate filament cytoskeleton, intermediate filament, keratin filament, and cornified envelope. Epidermal development, skin development, epidermal cell differentiation, keratinocyte differentiation, keratinization, and cornification were genes related to biological processes (BP). Based on KEGG enrichment analysis, we found the correlation between retinol metabolism, metabolism of xenobiotics by cytochrome P450, drug metabolism–cytochrome P450, *Staphylococcus aureus* infection, chemical carcinogenesis, IL-17 signaling pathway, galactose metabolism, and pentose and glucuronate interconversions with DEGs (Figures 7A–D). Furthermore, we analyzed the protein interactions between lncRNA ST7-AS1 associated genes through the String database to show the relationship between them in the interaction network (Figure 7E).

### GSEA Identifies a Signaling Pathway Related to LncRNA ST7-AS1

**FIGURE 8:**
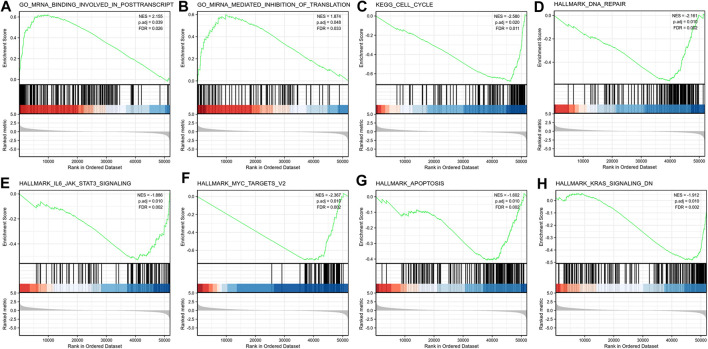
Enrichment plots from the GSEA. GSEA results showing **(A)** miRNA mediated inhibition of translation, **(B)** mRNA binding involved in posttranscription, **(C)** cell cycle, **(D)** DNA repair, **(E)** IL6 JAK STAT3 signaling, **(F)** MYC targets v2, **(G)** apoptosis, and **(H)** KRAS signaling.

We conducted GSEA between high lncRNA ST7-AS1 expression set and low lncRNA ST7-AS1 expression set to identify different signaling pathways in BRC, which revealed marked differences (FDR<0.25, adjusted *p* value < 0.05) in enrichments of MSigDB Collections (c2.cp.biocarta and h.all. v6.1 symbols). Based on normalized enrichment score (NES), the most significantly enriched signaling pathway was selected, as shown in Figure 8. In particular, lncRNA ST7-AS1 was related to miRNA mediated inhibition of translation, mRNA binding involved in posttranscription, cell cycle, DNA repair, IL6 JAK STAT3 signaling, MYC targets v2, apoptosis, and KRAS signaling.

### The Correlation Between LncRNA ST7-AS1 Expression and Immune Infiltration

**FIGURE 9:**
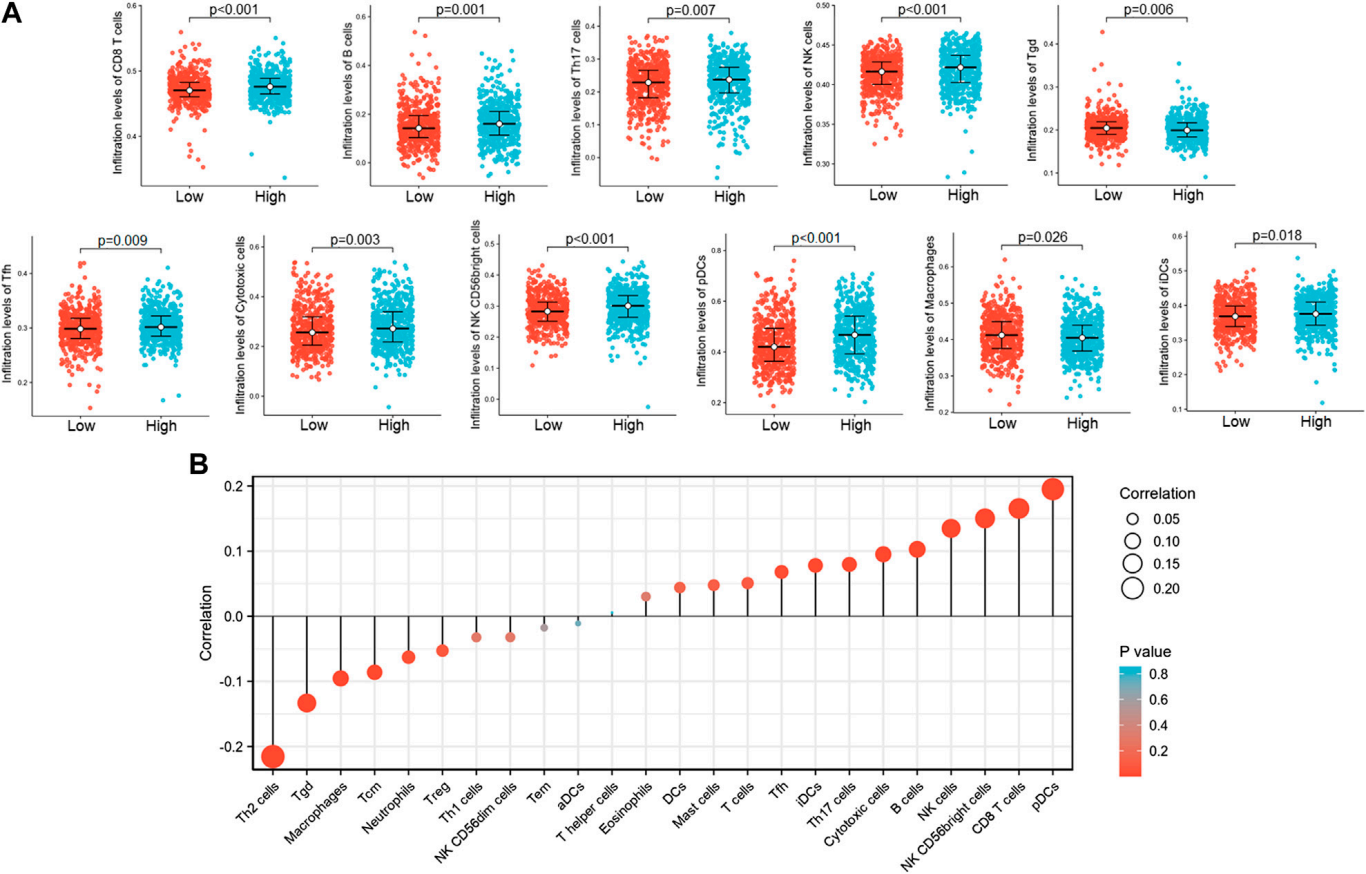
The lncRNA ST7-AS1 expression was correlated with immune infiltration in the tumor microenvironment. **(A)** Different proportions of immune cell subtypes in tumor samples in high and low lncRNA ST7-AS1 expression groups. **(B)** Correlation between the relative abundances of 24 immune cells and lncRNA ST7-AS1 expression level.

The Spearman correlation was used to show the correlation of ST7-AS1 expression level (TPM) with immune cell infiltration level quantified by ssGSEA in the BRC tumor microenvironment. We found that ST7-AS1 expression was negatively correlated with the abundances of immunocytes (Th2 cells, Tgd, macrophages, Tcm, etc.) and positively correlated with abundances of innate immunocytes (pDCs, NK cells, B cells, CD8 T cells, etc.) (Figure 9).

## Discussion

LncRNA is emerging as a tumor suppressor or oncogene in a number of tumors, as well as a potential therapeutic molecular target or biomarker with prognostic value (Cui et al., 2017). LncRNA ST7-antisense RNA 1 (AS1) is an antisense transcript for tumorigenicity 7 protein (ST7) suppressor located on 7q31.2 (Qin et al., 2019). LncRNA ST7-AS1 may influence the occurrence and development of laryngeal squamous cell carcinoma, but its biological function is entirely unknown, especially in the progression of cancer. As far as we know, there has been no study on lncRNA ST7-AS1 expression and its potential prognostic impact on BRC. This study focuses on the potential role of lncRNA ST7-AS1 in BRC.

Numerous studies have shown that lncRNA expression level plays an important part in the occurrence and progression of cancer (Yao et al., 2019). According to our bioinformatics analysis and high-throughput RNA sequencing data in TCGA, compared with normal tissue, BRC had a significantly lower expression level of lncRNA ST7-AS1. Decreased lncRNA ST7-AS1 expression in BRC was correlated with advanced clinical pathologic characteristics (high grade, histological type, age, menopause status, and HER2 status), survival time, and poor prognosis. Thus, our results suggested that lncRNA ST7-AS1 downregulation occurs in a number of BRC cases, and its role as a potential diagnostic and prognostic marker is worthy of further clinical validation.

In order to make a more accurate prognostic prediction, nomograms have been established, which had a better performance than conventional staging systems in some cancers. In our study, a nomogram was established using the results of multivariate Cox analysis which combined lncRNA ST7-AS1 with independent clinical risk factors (pathologic stage, ER status, and age). The calibration plot showed a good consistency between actual values and predicted values for 1-, 2-, and 3-year OS, DFI, and DSS. We constructed the model based on a complementary perspective of each tumor to provide personalized scores for individual patients.

There is another new finding from this study; that is, lncRNA ST7-AS1 participates in the cell cycle, apoptosis, and DNA repair. Genomic instability and mutagenesis belong to basic characteristics of cancer cells. Kinases and their associated signaling pathways conduce to the stabilization and repair of genomic DNA (Karimian et al., 2016; Yogosawa and Yoshida, 2018). LncRNA ST7-AS1 in BRC plays a role in regulating various signaling pathways related to inflammation, such as the IL6/JAK STAT3 signal pathway. It has been shown in previous studies that the IL-6/JAK STAT3 pathway is abnormally highly activated in many cancers, which is generally associated with poor clinical prognosis. In the tumor microenvironment, IL-6/JAK STAT3 signaling contributes to proliferation, invasiveness, and metastasis of tumor cells and strongly inhibits the antitumor immune response (Johnson et al., 2018). Moreover, STAT3 acts as the main mediator of RCC proliferation induced by IL-6 (Horiguchi et al., 2002).

Equally important, our study used ssGSEA and Spearman correlation to reveal the association between lncRNA ST7-AS1 expression and immune infiltration levels in BRC. We found the association between lncRNA ST7-AS1 and T helper cells and DC cells. Many tumors, including lung cancer, glioma, cervical cancer, BRC, gastric cancer, and colorectal cancer, all have Th1/Th2 balanced drift in the body, and Th2 cells are often dominant, which may be related to the immune escape of tumors (Shurin et al., 1999; Matsuzaki et al., 2005). Due to the relationship between Th1/Th2 balance drift and a variety of diseases, more and more research is tending to find and develop drugs and methods that can reverse or stabilize the Th1/Th2 balance. For example, the application of cytokines or cytokine antagonists to reverse Th1/Th2 balance drift in the treatment of tumors and other diseases: Th1 cytokines can promote the transformation of Th1/Th2 balance to Th1 state, while inhibiting the dominant expression of Th2. Th2 cytokines had the opposite effect (Hong et al., 2013; Saxena and Kaur, 2015). There is a close correlation between DC and tumor occurrence and development, and patients with a large number of infiltrating DC in most solid tumors have a good prognosis. The core of an effective antitumor immune response is to generate a cellular immune response dominated by CD8^+^ T cells, which are also the basis of DC as an immunotherapy (Huber et al., 2018).

Although the approach used in this study helps to understand the relationship between lncRNA ST7-AS1 and BRC, some limitations still exist. Firstly, to fully and comprehensively elucidate the specific role of lncRNA ST7-AS1 in BRC development, various clinical factors, such as details of patients’ treatments, should be considered. However, because the experiments are carried out in different laboratories, the public database is lack of such information or the treatments are inconsistent. Secondly, the number of healthy subjects used as controls differs significantly from that of cancer patients in this study, so further studies should be carried out to maintain a balanced sample size. In conclusion, limitations still exist in retrospective studies, especially the inconsistency of interventions and the lack of certain information. Therefore, future prospective studies are needed to eliminate analysis bias of this study which is retrospective in nature. Because this study was based on RNA sequencing data from the TCGA database, we could not elucidate the lncRNA ST7-AS1 expression at the protein level, nor could we clearly evaluate the direct mechanism by which lncRNA ST7-AS1 participates in the development of BRC. Therefore, the direct mechanism in BRC should be further studied.

## Conclusion

To conclude, our study showed decreased expression of lncRNA ST7-AS1 in BRC samples. Furthermore, low expression of lncRNA ST7-AS1 was associated with adverse outcomes. We also established a nomogram using lncRNA ST7-AS1 to predict 1-, 3-, or 5-year survival in patients with BRC. In terms of biological functions, we showed the involvement of lncRNA ST7-AS1 in cell cycle, DNA repair, and immune cell infiltration in the BRC immune microenvironment. We found the correlation of lncRNA ST7-AS1 with T helper cells and DC cells. In conclusion, these results indicate that lncRNA ST7-AS1 has a potential role in BRC as a prognostic marker and therapeutic target.

## Data Availability

Publicly available datasets were analyzed in this study. This data can be found here: The Cancer Genome Atlas (https://portal.gdc.cancer.gov/).
